# Coffee-ring formation through the use of the multi-ring mechanism guided by the self-assembly of magnetic nanoparticles

**DOI:** 10.1038/s41598-022-24521-x

**Published:** 2022-11-22

**Authors:** M. Marć, W. Wolak, A. Drzewiński, M. R. Dudek

**Affiliations:** Institute of Physics, University of Zielona Gora, ul. Szafrana 4a, 65-069 Zielona Gora, Poland

**Keywords:** Materials science, Nanoscience and technology

## Abstract

The control of the mechanism leading to the appearance of the ring-shaped stains from the dried liquid colloidal droplets has been the subject of intense studies over the last 25 years. This stems from the immense significance of this effect for technological applications. One of the key open topics in this field is the emergence of a regular multi-ring deposit from the dried droplet. Here, we show that magnetic nanoparticles in a drying magnetic liquid droplet can self-assemble into a multi-ring deposit structure, and even more importantly, a magnetic field can be turned on to control the underlying processes. The magnetic liquid is prepared as an aqueous suspension of Fe$_3$O$_4$ magnetic nanoparticles stabilized with (3-Aminopropyl)triethoxysilane (APTES) and its droplets are placed on low-density polyethylene (LDPE) film. The results of this work are expected to be very promising in the case of multiple applications including ink-jet printing methods and 2D printed electronics.

## Introduction

After the paper by Deegan et al.^1^ in 1997 about the formation of the ring-shaped deposit (the so-called “coffee-ring”) of the dried liquid colloidal droplets, thousands of publications related to this issue have appeared. The importance of this research for science and engineering was demonstrated by a short retrospective publication by Larson^2^ in the same journal 20 years later. However, it is worth noting that despite a very large number of publications, it is still possible to observe a very large number of new and constantly appearing studies focused on this topic. This stems from the fact that some of the fundamental mechanisms leading to the control over the coffee-ring formation are still not well-understood or can be further improved to enhance their efficiency and appeal from the point of view of commercial applications. One such example corresponds to the possibility of the appearance of the regularly spaced multi-ring deposit from dried colloidal droplets instead of a single irregular stain. This 2D feature of some droplet deposits is the subject of wide discussion both from the theoretical and experimental side^3–9^.

From the perspective of 2022, the recent research on the drying droplet focuses mainly on several related topics: control over the formation of “coffee-ring” deposits^5,10–14^, diagnostics and bioengineering^15,16^, film coating^17^, and ink-jet printing for 2D crystals and electronics^18,19^. It is a part of the broadly understood field of microfluidics including droplet dynamics, droplet shaping or space-separated chemical or photocatalytic reactions in the micro-droplet arrays^20–23^. In this work, we are concerned with the phenomenon of the multi-ring deposits from dried droplets. We show that magnetic nanoparticles can self-assemble into the regular 2D network of nanoparticle rings where the distance between these rings and the number of them can be controlled by applying a magnetic field gradient. In this study, magnetic liquid droplets with this property are placed on the hydrophobic low-density polyethylene (LDPE) film. This non-polar material, due to its chemical resistance and flexibility is used mainly for packaging, and insulation, but recently it shows an increasing role in biomedical applications^24^. The results obtained in this work also fit perfectly into the new class of proposed green electronics materials^25^.

**Figure 1:**
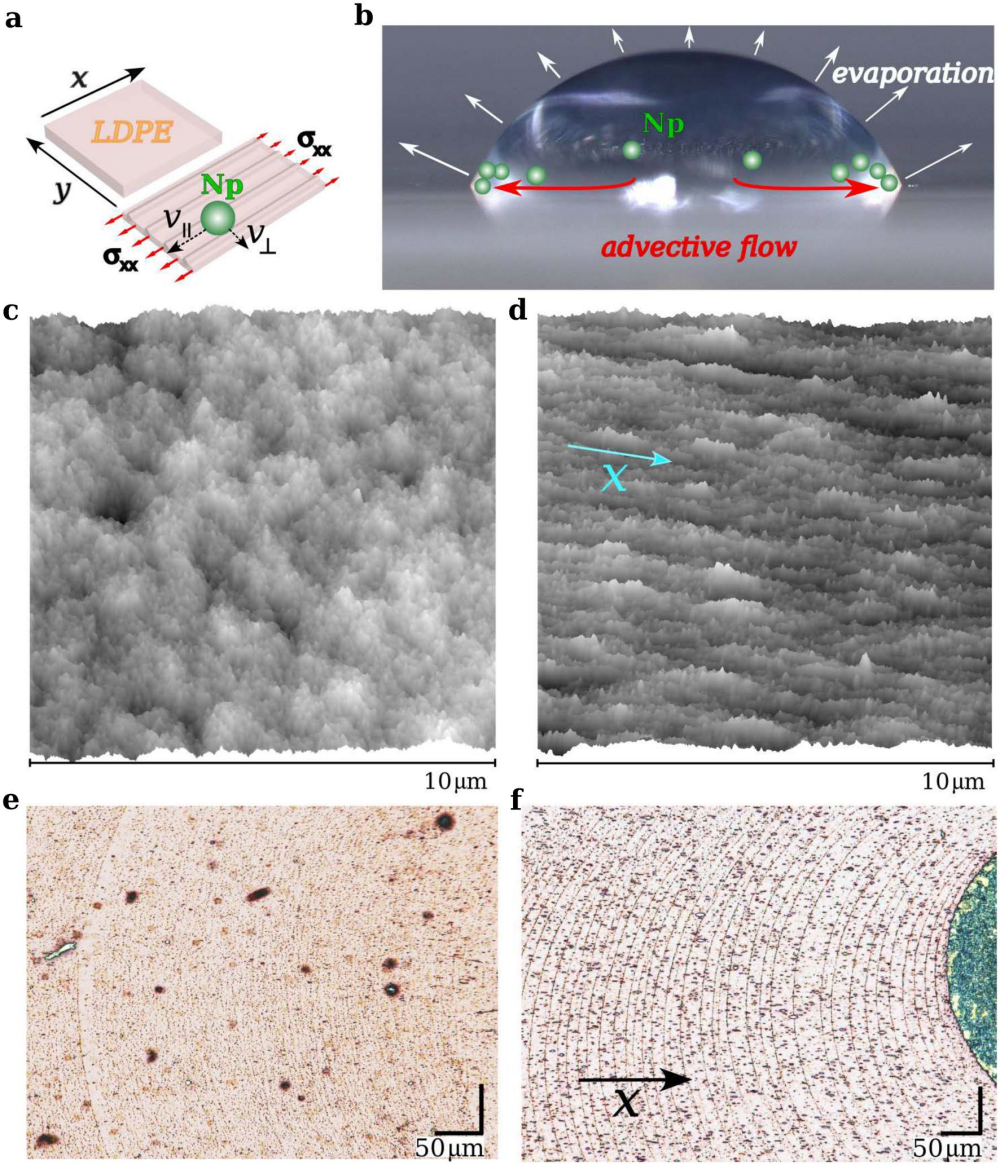
Scheme for obtaining multi-ring stains from a dried droplet of magnetic liquid on LDPE film. (**a**) Obtaining a stretched substrate after the application of normal stress $\sigma _{xx}$ in the x-axis direction. At the bottom of the droplet layer, the effective nanoparticle speed $v_{\perp } \ne v_{\parallel }$. (**b**) Evaporating droplet and magnetic nanoparticles (green symbols) in an advective flow due to the evaporation. (**c**) AFM image of the surface of the pristine LDPE. (**d**) AFM image of the stretched LDPE film surface where the original sample width of 1 cm was stretched to 5 cm. The arrow shows the direction of the *x*-axis in which the film was stretched. (**e**) Optical microscope image of the stains from the dried droplet on pristine LDPE. (**f**) Same as in (**e**) but on the stretched LDPE. The arrow shows the direction of stretching as on panel (**d**). The average height of the nanoparticle deposit, in panels (**e**) and (**f**), ranges from 30 to 100 nm. The optical visualization of them is possible due to the light scattering effect. This is also reason for the numerous dark spots visible outside the ring structures, which represent interior LDPE film irregularities.

## Results and discussion

### Sessile droplet of magnetic liquid

**Figure 2:**
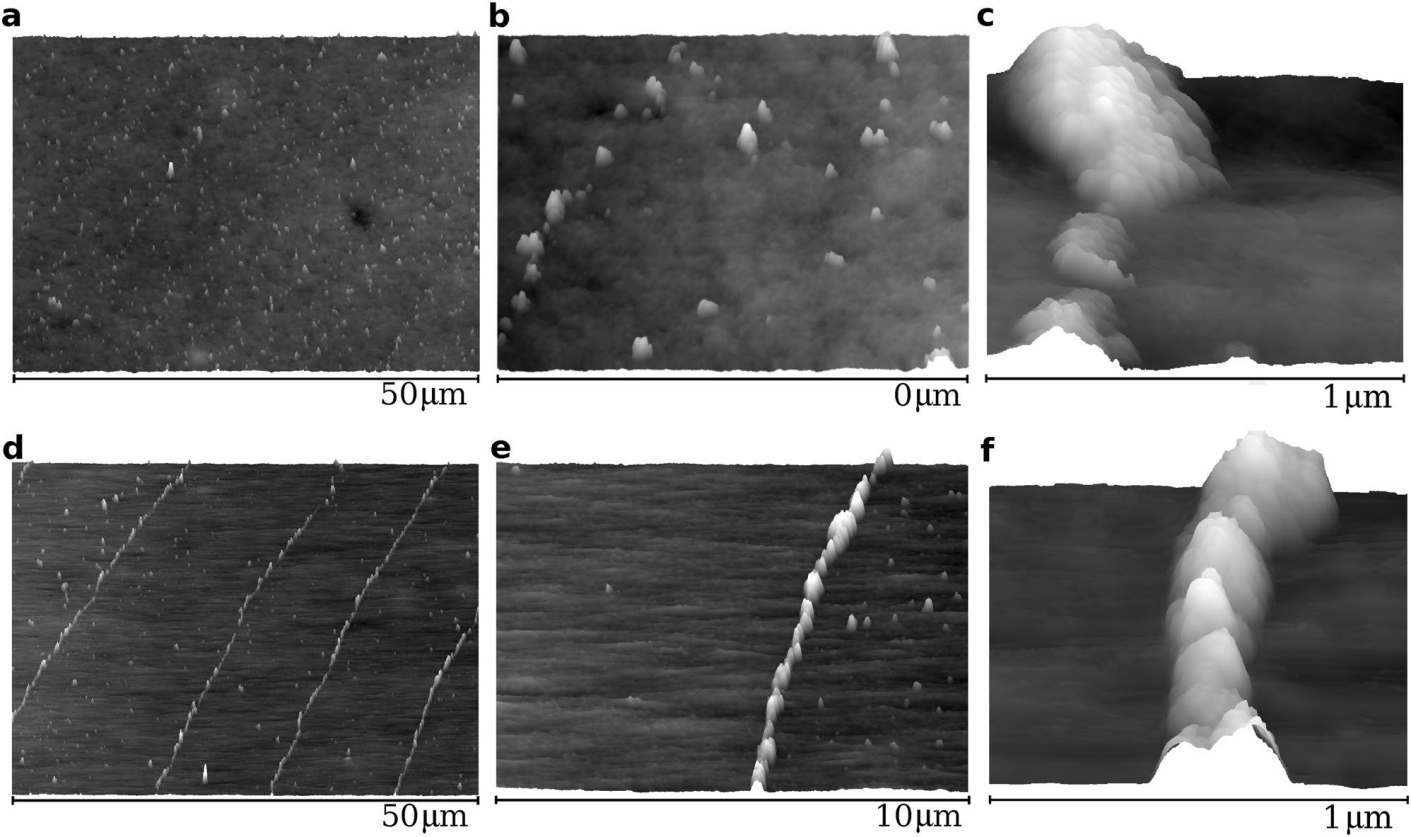
AFM topography of the representative fragments of the magnetic liquid droplet deposit on LDPE film. (**a**–**c**) deposit on pristine LDPE. (**d**–**f**) Deposit on stretched LDPE. In both types of LDPE, the average deposit height in panel (**c**) and (**f**) is about 60 nm.

In all experiments under discussion, 1 $\mu$L of a droplet of an acidic aqueous suspension (pH = 4) of magnetic nanoparticles Fe$_3$O$_4$ coated with (3-Aminopropyl)triethoxysilane (APTES) is placed on the LDPE film, as it has been shown in Fig. 1, and it starts to dry under the ambient conditions at a temperature around 25 $^\circ$ C and humidity around 34% (cf. “Methods” section for details of preparing the magnetic liquid). The initial diameter of the deposited droplet was about 2.7 mm. During the drying process, the contact line of the magnetic liquid droplet shrinks with some speed, it slows down when it is pinned to the substrate by the accumulated magnetic nanoparticles and at some time moment, it jumps to a new location leaving the ring-shaped deposit composed of the collected magnetic nanoparticles stuck to the substrate (Figs. 1e,f, 2a–f, cf. Movie 1 in Supplementary). The nanoparticle ring formation process then repeats itself with a new droplet radius *R*. The main reason for the accumulation of magnetic nanoparticles is the loss of droplet mass due to evaporation at the contact line which leads to the appearance of a liquid flow in the lower layer of the droplet to compensate for this loss and, consequently, an outward flux of nanoparticles that begin to accumulate at the contact line^1,3^. These nanoparticles become pinned to the substrate if their attraction to the substrate and the hydrodynamic drag forces balance the surface tension experienced by them at the contact line^7^ (cf. “Methods” section Pinning forces). In the case of magnetic nanoparticles, the application of an external magnetic field, e.g. by placing a magnet under a drying droplet, may result in fewer nanoparticles being sufficient to balance the surface tension (cf. Supplementary Fig. S4).

The quality of the resulting multi-ring structure strongly depends on the prepared substrate. We considered two cases of LDPE film surface, its pristine form as in Fig. 1c and the stretched form (sample width of 1 cm was stretched to 5 cm) as in Fig. 1d. The stretched film becomes strongly asymmetric in the stretching direction (the *x*-axis direction in Fig. 1a,d,f). In the sample from Fig. 1d, the root mean square roughness of the height deviations measured from their mean value in the stretching direction is equal to 4.87 nm and 6.59 nm in the transverse direction. This has consequences for the shape of the nanoparticle rings because the effective viscosity of the nanoparticles approaching the contact line starts to depend on the moving direction. Note that the difference in roughness in the *x*-direction and the *y*-direction is almost 2 nm, while the magnetic nanoparticles coated with the APTES layer have a size of about 30 nm (cf. “Methods” section). We can observe very clear continuous ring-shaped deposit structures in Figs. 1f and 2d but this is not the case in Figs. 1e and 2a for the droplet drying on the pristine LDPE (cf. Supplementary Fig. S2). However, it should be added, that the nanoparticle rings in Fig. 1f become disrupted in the droplet areas located in the direction perpendicular to the stretching direction (cf. Supplementary Fig. S1).

To obtain the ring structures, the quality of the magnetic liquid is also important. The first task of APTES coating on magnetic nanoparticles is to prevent agglomeration of magnetic nanoparticles due to the dipole-dipole interactions. In the acidic solution with pH = 4, the coated nanoparticles acquire a positive surface charge—the value of the zeta potential is around 20.0 $\times$ 10$^{-3}$ V^26^. Then, the Coulomb repulsion between the nanoparticles stabilizes the liquid colloid. At this value of pH, the zeta potential of LDPE film is negative and its value is around $-25.0\times 10^{-3}$ V^27^. This means the presence of the strong electrostatic attraction of the coated nanoparticles with the substrate at distances smaller than the Debye length which is equal to $\lambda _D \approx 43$ nm at pH = 4. More, dissociated amine groups from APTES cause the hydrophobic LDPE substrate to change to hydrophilic over the areas where the nanoparticles are pinned to the substrate, in particular at the contact line of the drying droplet.

Different results for nanoparticle multi-ring structures for two substrates representing the stretched LDPE and the unstretched one, among others, in Fig. 2a,b suggest that the directional patterning of the substrate introduces different rules both for the nanoparticles moving in the lower part of the drying droplet and at the contact line. The effects of the patterned substrate on the particle deposition were discussed e.g. in the papers^14,28,29^.

### Effect of the applied external magnetic field

**Figure 3:**
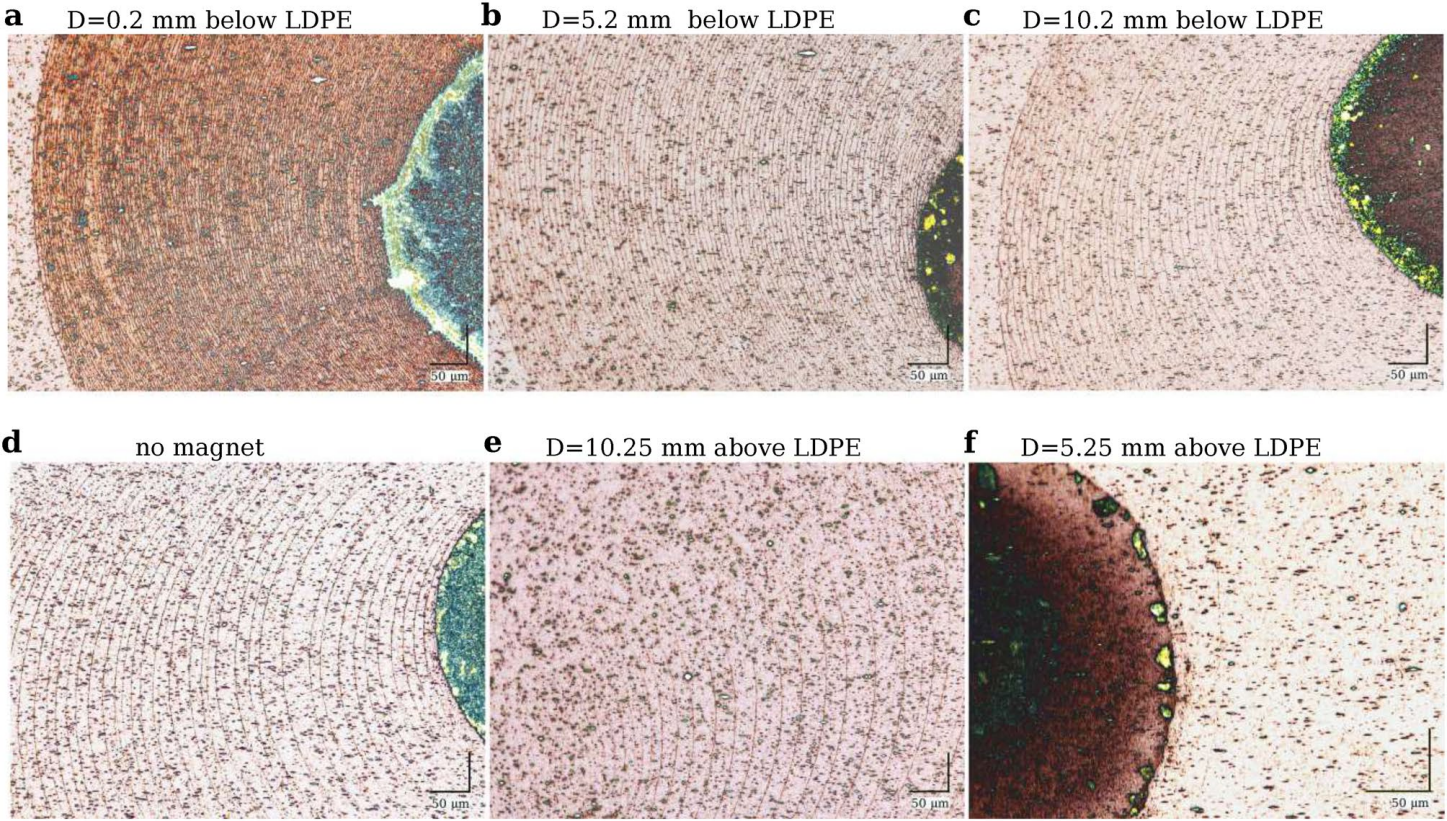
Optical microscope images of the stains from magnetic liquid droplets dried on the stretched LDPE film in the presence of the magnetic field gradient. The cylindrical neodymium magnet with the residual magnetic flux density $B_r \approx$1T was used. Different distances *D* of the magnet from the LDPE film, on which the drying droplet was placed, make it possible to control the concentration of magnetic nanoparticles in the lower part of the droplet as a result of their movement in a magnetic field gradient towards the magnet. (**a**) $D=0.2$ mm below the film. (**b**) $D=5.2$ mm below the film. (**c**) $D=10.2$ mm below the film. (**d**) No magnet. (**e**) $D=10.25$ mm above the film. (**f**) $D=5.25$ mm above the film. The optical visualization of the magnetic nanoparticle rings is due to the light scattering effect. Numerous dark spots outside the ring structures result from the light scattering on interior LDPE film irregularities.

If a magnet is placed below the droplet, an additional drift of magnetic nanoparticles appears towards it, increasing the concentration of nanoparticles in the lower layer of the drying droplet. Thus, the flux of nanoparticles moving towards the contact line increases. If, on the other hand, the magnet is placed above the droplet, the nanoparticles move to the upper part of the droplet, reducing their concentration in the lower droplet layer and, consequently, the flux of nanoparticles towards the contact line. Hence, the distances between the rings of the deposit can change depending on the magnetic field. The panels in Fig. 3a–f prove that an external magnetic field can control the deposit from a dried droplet.

**Figure 4:**
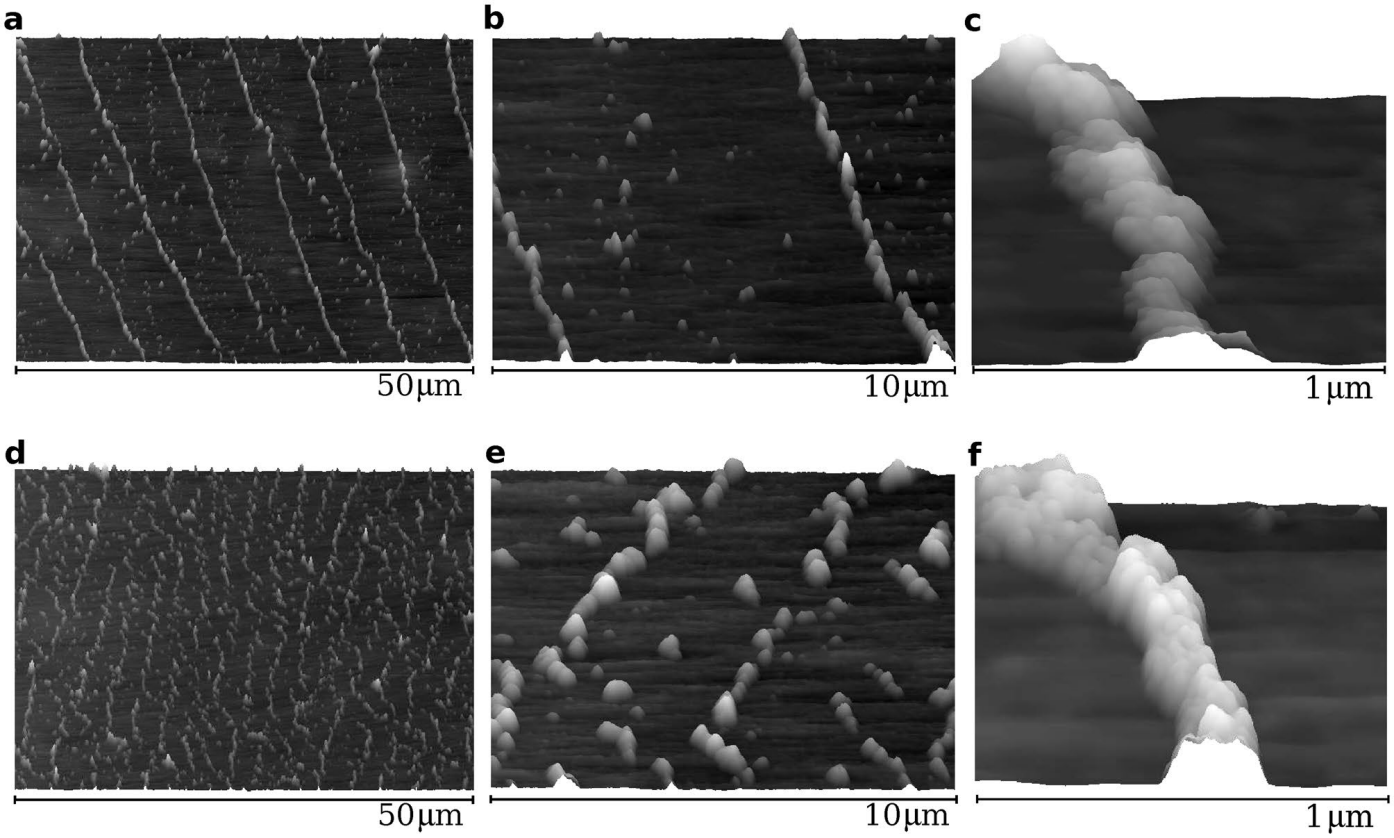
AFM topography of multi-ring deposit from a dried droplet on the stretched LDPE film in the case of a magnet below the film. (**a**–**c**) $D=5.2$ mm. (**d**–**f**) $D=0.2$ mm. The deposit height in panels (**c**) and (**f**) does not exceed 60 nm.

In the case of too strong magnetic field, the nanoparticle ring structures begin to become deformed due to the surface roughness, as in Fig. 4a–f. Nevertheless, they remain much more ordered compared to the analogous case for a droplet drying on a pristine LDPE (cf. Supplementary Fig. S2).

**Figure 5:**
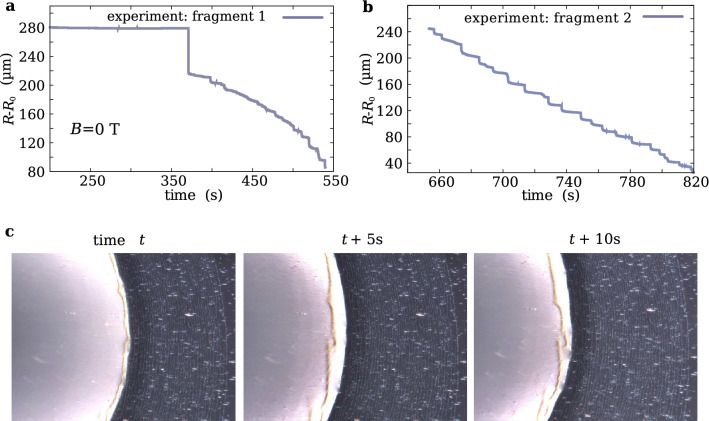
Evolution over time of a drying droplet. (**a**) The plot of the droplet radius *R* vs. time data obtained with the help of the optical microscope measurements along the stretching direction of the LDPE film (no magnetic field, *B* = 0 T). The $R_0$ point is the reference point under the microscope camera objective against which the $R-R_0$ distance was measured. (**b**) Continuation of the measurement (**a**) from another reference point $R_0$ after the microscope camera reached the edge of the measurement window. (**c**) The frames from a movie under an optical microscope, where the fragments of the nanoparticle ring-shaped deposits float. The droplet dries in the presence of a magnet placed 5.2 mm below the LDPE film.

In the experiments leading to the results presented in Figs. 3, 4 and 5 a cylindrical NdFeB magnet with the radius $R=4.5$ mm, the height $L=4.8$ mm, and the residual magnetic flux density $B_r \approx 1$T was used. To estimate the $B_r$ value of this magnet, the following approximate formula was used for the dependence of the magnetic field strength *B* on the distance *D* from the magnet along the *z*-direction (cylinder axis direction)^30^:

1 $$\begin{aligned} B(D)=\frac{B_r}{2} \left( {\frac{L+D}{\sqrt{(L+D)^2+R^2}}-\frac{D}{\sqrt{D^2+R^2}}} \right) , \end{aligned}$$

where the value of $B_r$ was optimized with respect to the relationship $B=B(D)$ obtained by measurements with a magnetometer (Gauss-/Teslameter FH 54 (Magnet-Physik GmbH)).

### Drying droplet kinetics

In Fig. 5a,b, the typical dependence of the droplet radius *R* on time has been presented in the case when there is no magnetic field (*B* = 0 T). It suggests that the average time between the succeeding jumps of the radius *R* over distances larger than 5 μm can be as short as a few seconds, e.g. 8 s in the case of Fig. 5b. This time interval can be shortened several times in the presence of the magnetic field of a magnet placed under the droplet. However, in all these cases we observed that the heights of the nanoparticle ring structures are of the same order of magnitude. This is the effect of the capillary forces which can detach ring fragments with the nanoparticle agglomerates when their elastic deformation exceeds some threshold value. This is suggested by Fig. 5c, where three frames taken from the movie (Movie 2 in Supplementary) show the event with the sudden appearance of the floating ring-shaped fragments after jumping off the contact line to a new location with the smaller radius *R*. In the case of APTES material, the shear modulus $G \approx 1.6 \times 10^6$ Pa and the horizontal capillary force per unit length, $\gamma \cos (\theta )$, where $\gamma$ is the water solution surface tension, can introduce the elastic deformation $\delta$ of the ring, which can be approximated by the Hook’s law, $\delta \approx \frac{\gamma }{G} \cos (\theta ) = 7.8$ nm for the droplet contact angle $\theta =80^{\circ }$. Some detached fragments of the ring structure can also originate from the areas of a drying droplet where the contracting contact line becomes roughly parallel to the LDPE film stretching direction.

### Computer simulation

**Figure 6:**
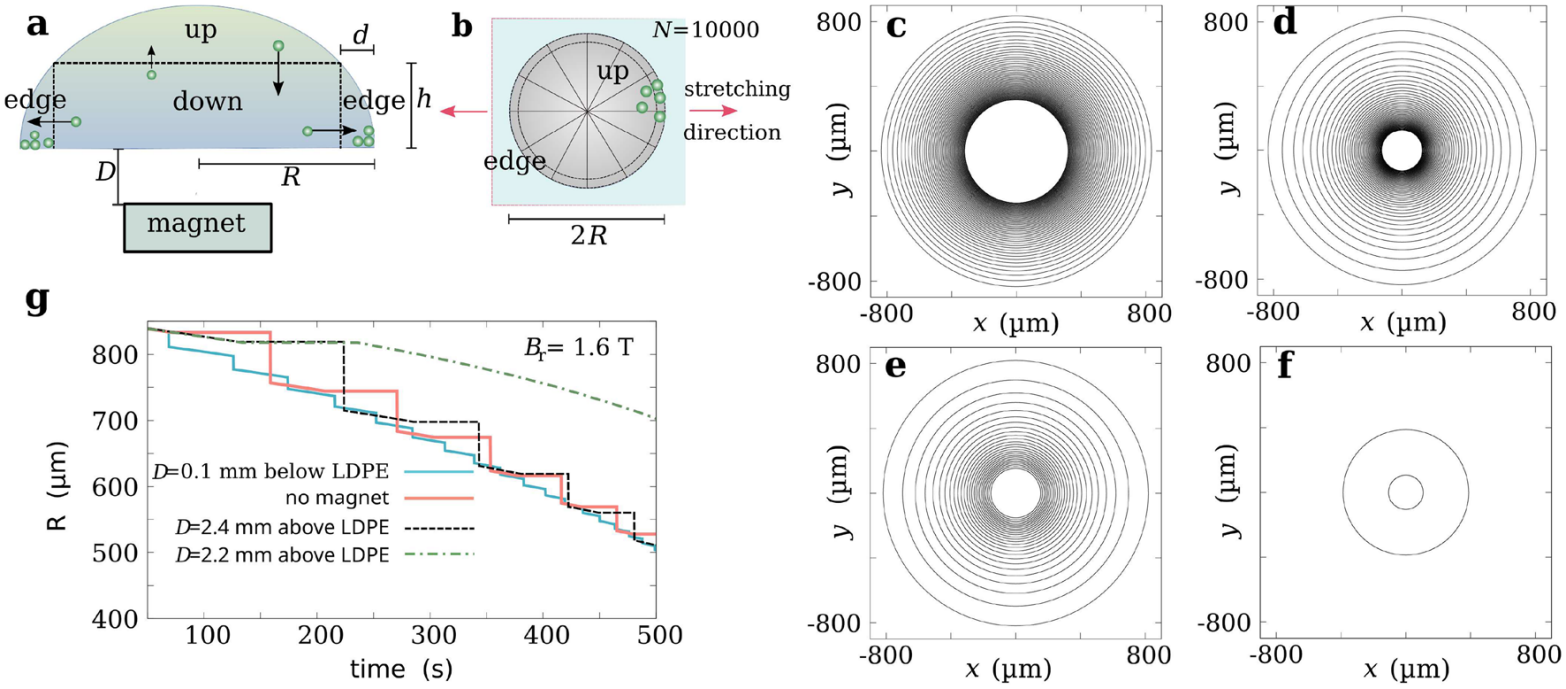
Theoretical model scheme and results. (**a**) Cross-section of a magnetic liquid droplet with a coarse-grained division into two layers: the lower part with the “down” area and the “edge” area, where the outward drift of magnetic nanoparticles takes place due to the droplet evaporation, and the upper part representing the “up” area. When a magnet is located below the droplet an extra drift of magnetic nanoparticles from the area “up” to “down” is present, otherwise, for the magnet above the droplet, the nanoparticle drift from “down” to “up” is present. (**b**) Division of the droplet volume into N units with the effective viscosity at the bottom layer that depends on the orientation concerning for the stretching direction. (**c**) Multi-ring deposit for a magnet at distance $D=0.1$ mm below the LDPE film, (**d**) Multi-ring deposit without the presence of a magnet, (**e**) Multi-ring deposit for a magnet at distance $D=2.4$ mm above the LDPE film, (**f**) Same as for case (**e**) but for $D=2.2$ mm, (**g**) Dependence of radius *R* on time. Some parameters: *T* = 300 K, humidity = 0.34, pH = 4, $\eta$ = 0.001 Pa s, $\gamma$ = 0.072 N/m, $B_r=1.6$ T.

The presentation of the experimental results concerning the deposit of the drying droplet in the form of a series of rings made of magnetic nanoparticles requires supplementation from the theoretical model of the occurring phenomenon. For a qualitative analysis of the nanoparticles pinned at the contact line the phenomenological approach as in the papers^1,4,7^ is sufficient. We have extended this approach to magnetic nanoparticles. In Fig. 6a,b, the scheme of the model is shown, and its main goal is to balance two flows of magnetic nanoparticles, the flow to the edge of the droplet (“down” $\rightarrow$ “edge”) due to the loss of droplet mass caused by the droplet evaporation and the downward flow of nanoparticles in the magnetic field gradient in the case of a neodymium magnet placed under the droplet (“up” $\rightarrow$ “down”) or the upward flow for the magnet above the droplet (“down” $\rightarrow$ “up”). The results of the computer simulations presented in Fig. 6g are for the case of a magnet with $B_r$ = 1.6T. This allowed to emphasize the influence of the magnetic field gradient qualitatively in line with the experimental results. As in the paper^4^, the breadth *d* of the edge is a small value (in our case, the initial value of *d* is equal to $0.4 \mu$m) but it decreases in time accordingly to the droplet radius *R*. In the model, the height *h* of the segment “down” represents the level of the stagnation point of a droplet and it is equal to the droplet height at radial distance $r=R-d$ from the droplet center. By analogy to^4^, the mass fraction of nanoparticles $\Delta f_J(t)$ that flows during $\Delta t$ from the segment “down” to “edge” reads as the following:

2 $$\begin{aligned} \Delta f_J(t)=f_\mathrm{down} \frac{v_J \Delta t}{d}, \end{aligned}$$

where $v_J$ is the radial velocity of the local advective mass flow due to droplet evaporation at the area of the contact line^1,4^. Details on the expression on $\Delta f_J$ are in the “Methods” section. In our model, in the case of a magnet placed below (above) the droplet there is also some mass fraction of nanoparticles $\Delta f_M(t)$ that flows from the segment “up” to “down” (“down” to “up”) due to the vertical component (*z*-direction) of the magnetic force, $\overrightarrow{F_M}=-(\overrightarrow{m} \cdot \overrightarrow{\nabla }) \overrightarrow{B}$, acting on the nanoparticles with the magnetic moment $\overrightarrow{m}$ and it reads as the following:

3 $$\begin{aligned} \Delta f_M(t)=f_{\mathrm{up (down)}} \frac{v_M \Delta t}{h}, \end{aligned}$$

where

4 $$\begin{aligned} v_M=\frac{(\overrightarrow{F}_M)_z}{6 \pi \eta R_\mathrm{g}} \end{aligned}$$

is the velocity of a spherical magnetic nanoparticle coated with APTES in a viscous liquid of the viscosity $\eta$, and $R_g=R_\mathrm{NP}+d_\mathrm{A}$ is the total nanoparticle radius, where $R_\mathrm{NP}$ is the radius of the bare magnetic nanoparticle, and $d_\mathrm{A}$ is the thickness of the APTES layer. In the model, we assume the magnetic field B=B(z) as in Eq. (1). In this case, $(\overrightarrow{F}_M)_z=-m_z\frac{\partial B(z)}{\partial z}$, and we also assume that $m_z=M_\mathrm{sat} V_\mathrm{NP}$ is the magnetic moment of the nanoparticle with radius $R_\mathrm{NP}$, where the volume is equal to $V_\mathrm{NP}=4/3 \pi R_\mathrm{NP}^3$ and $M_\mathrm{sat}$ denotes magnetization ($4.45 \times 10^5$ A/m for magnetite). In the model, $R_\mathrm{NP}$ = 7 nm and $d_\mathrm{A}=10$ nm, and the magnetic dipole-dipole interactions are ignored.

The effect of LDPE film asymmetry due to its stretching was included by the division of droplet volume as in Fig. 6b into *N* subvolumes, the units representing the radial directions, in a coarse-grained way, along which the outward flow of magnetic nanoparticles takes place. This made it possible to provide each unit of “edge” with an effective value of $v_J$ (Eq. (2)) that depends on the substrate roughness in the corresponding radial direction.

To simplify the model, the concentration of the magnetic liquid is assumed to be uniform in the volumes “up” and “down”. The computer simulation results show, the contact line that moves with different speeds depending on if it is pinned on hydrophilic nanoparticle agglomerates or it is shrinking on the hydrophobic substrate in the constant angle mode (Fig. 6g). During the contact line becomes pinned, only a small drift of the droplet radius *R* is present. The presence of such a drift was suggested in^3^. In our model, this drift is modeled using the Metropolis Monte Carlo method. It is sampling two modes of droplet behavior, the mode when the droplet radius *R* is constant and the contact angle $\theta$ decreases due to evaporation and the mode when during $\Delta t$ a small increase in $\theta$ and the respective decrease in *R* with the preserved droplet volume takes place. In the model, the contact line is depinning when the shear deformation of the nanoparticle ring exceeds a given threshold value and the contact line jumps to a new location with the smaller radius $R(t+\Delta t)$ and larger $\theta (t+\Delta t)$. It is assumed that the droplet volume does not change during the jump, i.e. $V(R(t),\theta (t))=V(R(t+\Delta t),\theta (t+\Delta t))$.

The computer simulation results in Fig. 6c–g support the finding from the experimental data that magnetic nanoparticles can form multi-ring deposits and that the external magnetic field can control the spacing between the rings. In Supplementary Fig. S5, the results concerning the evolution over time of the angle of the contact line are provided. They suggest a much smaller decrease of the contact angle for magnetic liquid droplets drying in the magnetic field from the magnet below the droplet than without the field or the field above the droplet.

The applied computer simulation schematic is the following:

(i) Set the initial parameters for the magnetic liquid droplet drying, $t=$0s, *R*(*t*)—droplet radius, $\theta (t)$—contact angle, $\theta _0$—Gibbs equilibrium contact angle, $C_\mathrm{up}(t), C_\mathrm{down}(t), C_\mathrm{edge}(t)$—volume “up”, “down”, and “edge” concentrations of magnetic nanoparticles, temperature *T*, and all parameters determining environmental conditions for droplet drying and the ones to calculate the pinning forces on the contact line.
(ii) Check number of magnetic nanoparticles at contact line (in the volume “edge”), calculate the net pinning force $F_p$ (see “Methods” section, and^7^) of the nanoparticles against the surface tension $F_c=2\pi R \gamma \cos (\theta )$.
(iii) If $F_p > F_c$, the pinning takes place. The droplet dries with decreasing $\theta$, as in the constant radius mode (CR mode), i.e. $\theta (t+\Delta t)=\theta (t)- \Delta \theta _\mathrm{CR}$ and $R(t)=R_\mathrm{CR}$ = const, or this value is corrected to $\theta (t+\Delta t)=\theta (t)- \Delta \theta _\mathrm{CR}+\Delta \theta$, where $\Delta \theta$ takes a small random value due to the slight decreasing drift of the radius $R(t+\Delta t)=R(t) - \Delta R$ taken place at constant volume. The Metropolis Monte Carlo algorithm is used to calculate the probability for this drift in *R*, $\textrm{Prob}=\textrm{min}(1,\textrm{e}^{-\Delta G/k_BT})$, where $\Delta G = G(R(t)-\Delta R,\theta (t)- \Delta \theta _\mathrm{CR}+\Delta \theta )-G(R_\mathrm{CR},\theta (t)-\Delta \theta _\mathrm{CR})$ is the droplet Gibbs free energy difference. The Gibbs free energy is defined as in^3^. If the horizontal deformation of the magnetic agglomerates pinned at the contact line exceeds a given threshold value, the contact line detaches and takes a new value of *R* calculated from comparing the droplet volumes before a jump and after the jump.
(iv) Adjust the changes in volumes “up”, “down”, “edge” of droplet for the new values of $R(t+\Delta t)$ and $\theta (t+\Delta t)$, as well as concentrations $C_\mathrm{up}(t), C_\mathrm{down}(t), C_\mathrm{edge}(t)$ of nanoparticles and the respective nanoparticle flows between these volumes.
(v) Go to [(ii)] until the end condition is fulfilled.

## Conclusions

In this work, we show that the deposit from a dried magnetic liquid droplet can self-assemble into regular ring structures where the number of rings and the distance between them can be controlled by applying an appropriate magnetic field gradient. In this case, the magnetic liquid is a stable water colloid of the ultra-small magnetite nanoparticles coated with APTES. The colloidal stability of the magnetic liquid is ensured by the choice of pH = 4. The asymmetry of the hydrophobic LDPE film substrate, which appears after applying an appropriate uniaxial tensile stress, turns out to be sufficient for the appearance of a regular 2D sequence of the ring-like structures of the magnetic nanoparticle deposit. It should be added that in such a process, after the formation of the multi-ring droplet deposit, there is always a certain fraction of nanoparticles arranged in the form of a homogeneous spot. This spot can be removed in the final stage of the droplet drying process, but this is not the subject of this article. The possibility of programming the distance between the magnetic rings and the use of magnetic nanoparticles can be promising for 2D printing electronics. This property can also be promising for biotechnological applications due to the possibility of the functionalization of the nanoparticle rings, e.g. for different kinds of bioassays and molecular chip technologies.

## Methods

### Synthesis of Fe$_3$O$_4$ magnetic nanoparticles

The amount of 3.81 g of FeSO$_4$ $\cdot$ 7H$_2$O was dissolved in 100 ml of 0.02 M hydrochloric acid aqueous solution and the 7.41 g of FeCl$_3$ $\cdot$ 6H$_2$O was dissolved in 100 ml of distilled water. Solutions were mixed in a double neck round bottom flask and stirred at 400 rpm for 50 min in a nitrogen atmosphere. Next, the rotational speed of the mechanical stirrer was set to 800 rpm and 25 ml of a 25% ammonia solution was added dropwise (1 drop/s). Mixing was continued for 30 min at a speed of 800 rpm. Then, the appropriate amount of the suspension was taken and the washing procedure was carried out with distilled water.A neodymium magnet was used to separate nanoparticles from the suspension during the washing procedure. The mean size of the bare magnetic nanoparticles is about 12 nm and the standard deviation is about 3 nm.

### Coating with APTES

The amount of 4 ml of the prepared Fe$_3$O$_4$ magnetic nanoparticle suspension was taken, preceded by 15 min sonication. Next, 36 ml of ethanol (99.8%) was added to the suspension and sonicated for 15 min at a temperature 70 $^{\circ }$C. During sonication, mechanical stirring (1200 rpm) and at a temperature 70 $^{\circ }$C, the amount of 0.5 ml of 3-aminopropyltriethoxysilane (APTES, 99%) was added to the suspension. Stirring was continued for 2.5 hours. Subsequently, the washing procedure was conducted three times with ethanol and water, using a neodymium magnet to separate particles from the suspension. (Final volume at this point is 25 ml, suspension concentration 2.26 mg/ml.) Prior to the experiment the concentration was lowered and the appropriate amount of 2 M HCl was added to set the value of pH to be equal to 4. Final concentration of Fe$_3$O$_4$ magnetic nanoparticles was of 0.045 mg/ml.

### Magnetic liquid

The stable colloidal solution of magnetic liquid at pH = 4 was prepared with the Fe$_3$O$_4$ magnetic nanoparticle mass concentration 0.045 mg/1 ml where APTES was used to stabilize the solution. The Atomic Force Microscope (AFM) measurements suggested that the mean size of the magnetic nanoparticles coated with APTES used in our experiments was about 30 nm. One of the reasons for this size compared to 12 nm of the bare magnetic nanoparticles is that sonication of an aqueous suspension of the bare magnetic nanoparticles before coating with APTES leaves a fraction of the small nanoparticle agglomerations. The transmission electron microscope (TEM) exemplary images of the APTES-coated agglomerates have been shown in Supplementary Fig. S3.

### Velocity calculation

In the computer simulation, the total fraction of the mass per volume, $\Delta f_J(t)$, that is transferred from the droplet segment “down” to “edge” during $\Delta t$ is calculated separately in each subvolume unit ($i=1, 2, \ldots , N$) and the resulting values are summed up as follows:

5 $$\begin{aligned} \Delta f_J(t)=\sum _{i=1}^{N} f_\mathrm{down}^{(i)} \frac{v_J^{(i)} \Delta t}{d}, \end{aligned}$$

where $v_J^{(i)}$ is calculated with the help of the droplet evaporation current *J*. The expression for *J* can be found in papers^1,4^ and it reads as:

6 $$\begin{aligned} J(r)=J_0 (1-r^2/R^2)^{-\lambda }, \end{aligned}$$

where *r* denotes the radial distance from the droplet center, $J_0$ is the value of this current at the droplet center, and $\lambda =0.5 - \theta /{\pi }$. The corresponding speed of the mass flow in the droplet is equal to:

7 $$\begin{aligned} v_J=J/{\rho _\mathrm{liq}} \sin (\theta )), \end{aligned}$$

where $\rho _\mathrm{liq}$ is the liquid density. In Eq. (5), this speed is modified to include the directional substrate roughness of the unit *i* as follows:

8 $$\begin{aligned} v_J^{(i)}=v_J \sqrt{\left( v_{\parallel }^{(i)} v_{\parallel }^{(i)}+v_{\perp }^{(i)} v_{\perp }^{(i)}\right) }, \end{aligned}$$

where $v_{\parallel }^{(i)}=\cos (\alpha _i)$, $v_{\perp }^{(i)}= \kappa \sin (\alpha _i)$, and $\alpha _i$ represents the angle which the moving nanoparticle makes with the *x*-direction (Fig. 1), and $\kappa$ is the ratio of the substrate roughness along the *y*-direction and *x*-direction, respectively.

In Eq. (3), the magnetic field strength $B=B(z)$ at distance *z* from the magnet is calculated for the cylindrical neodymium magnet with the residual magnetic flux density $B_r=1.6$T.

### Pinning forces

In the computer simulation model, all $N_{\mathrm{NP}}^{\textrm{edge}}$ magnetic nanoparticles which have been collected in the segment “edge” are taken into account to calculate the total pinning force $F_p$ required to balance the capillary force $F_c$. It is assumed that $F_p=N_{\mathrm{NP}}^{\textrm{edge}} \left( {f (F_\mathrm{M}+F_\mathrm{DE}+F_\mathrm{VdW})+F_\mathrm{drag}} \right)$, where *f* represents the friction coefficient ($f=0.9$), $F_\mathrm{M}$ is the magnetic force strength (positive for magnet below the droplet and negative otherwise), $F_\mathrm{VdW}$ is the Van der Waals force ($F_\mathrm{VdW}(D)=-A R_\mathrm{NP}/6D^2$ in the geometry “sphere-flat surface” at distance *D*, and we set the Hamaker constant $A=71 \times 10^{-20}$J.), $F_\mathrm{DE}$ (as in^7^) is the electrostatic force between the nanoparticle and LDPE substrate, and $F_\mathrm{drag}$ (as in^7^).

## Supplementary Information

Supplementary Information.

Supplementary Video 1.

Supplementary Video 2.

## Data Availability

The datasets generated during and/or analysed during the current study are available from the corresponding author on reasonable request.
