# Supplementary material for: Coffee-ring formation through the use of the multi-ring mechanism guided by the self-assembly of magnetic nanoparticles
Source: Sci Rep. 2022 Nov 22;12:20131. doi: 10.1038/s41598-022-24521-x (PMC9684494; doi:10.1038/s41598-022-24521-x)
Supplement: Supplementary file 1 — Supplementary Information. [file 41598_2022_24521_MOESM1_ESM.pdf]

Supplementary Information for

Coffee-ring formation through the use of the multi-ring mechanism guided by the self-assembly of magnetic nanoparticles

M. Marć, W. Wolak, A. Drzewiński, M.R. Dudek

Correspondence to: M.Dudek@if.uz.zgora.pl

The Supplementary pdf file contains five figures: Figure S1, Figure S2, Figure S3, Figure S4, and Figure S5.

Complementary to the experimental data of magnetic liquid droplet on stretched LDPE film.

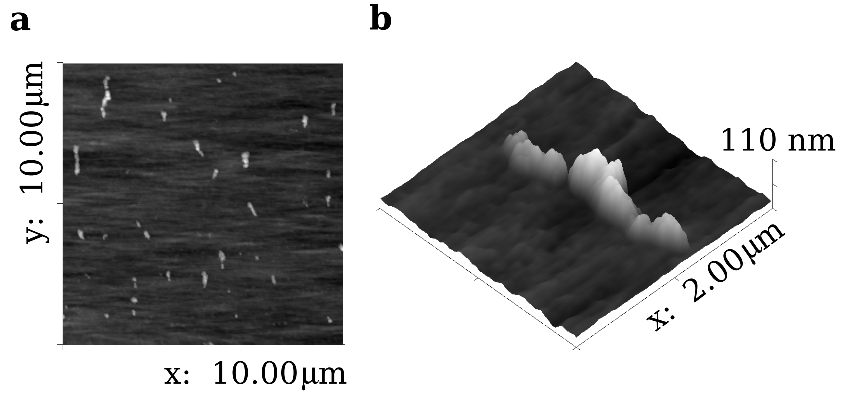

Figure S1 : **AFM topography of nanoparticle deposit from dried magnetic liquid droplet on stretched LDPE film in the y-direction. This direction is vertical to the stretching direction. a** Representative image corresponding to the 10 μm $\times$ 10 μm area. **b** The enlarged fragment from **a**.

In the main text of our work, we show that magnetic liquid deposits on LDPE film can take a regular multi-ring form which can be controlled with the help of

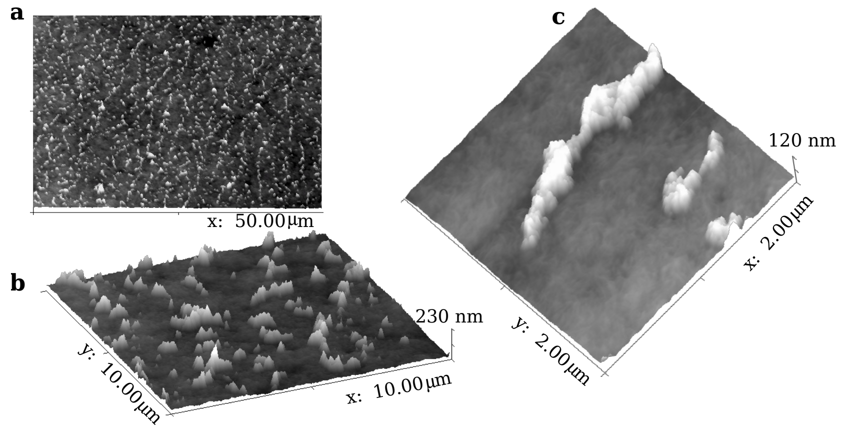

Figure S2 : **AFM topography of nanoparticle deposit from a dried magnetic liquid droplet on pristine LDPE film in a magnetic field gradient** (magnet 0.2 mm below LDPE film). **a** Representative image of nanoparticle deposit corresponding to the 50 μm $\times$ 50 μm area with the nanoparticle agglomerates. **b** The enlarged fragment from **a**. **c** The enlarged fragment of **b**.

the external magnetic field. In this Supplementary, in Figure S1, we show the fragments of the corresponding nanoparticle deposit when the contact line of a drying magnetic liquid droplet is roughly parallel to the stretching direction of the LDPE film (tensile stress is applied to LDPE, which introduces a strongly asymmetric surface of the film, cf. the work). In the figure, the x-direction is approximately the LDPE film stretching direction. The increased roughness of the LDPE film in the y-direction slows down the outward flow of the magnetic nanoparticles, and consequently, the number of particles reaching the contact line is smaller. Then, the nanoparticle agglomerates which are pinned at the contact line are too small to prevent the contraction of the whole contact line during droplet evaporation. They have only a local pinning effect on the contact line. In the figure, it is evident that they are dispersed over LDPE film just as they were being pulled by capillary forces.

In Figure S2, the structure of droplet deposit on the pristine LDPE film in a strong magnetic field has been shown. Notice, that this structure is very irregular compared to the droplet deposits on asymmetric LDPE film (cf. Fig 4d in the Main text of our work).

In Figure S3, the TEM images of the coated magnetic nanoparticles have been shown.

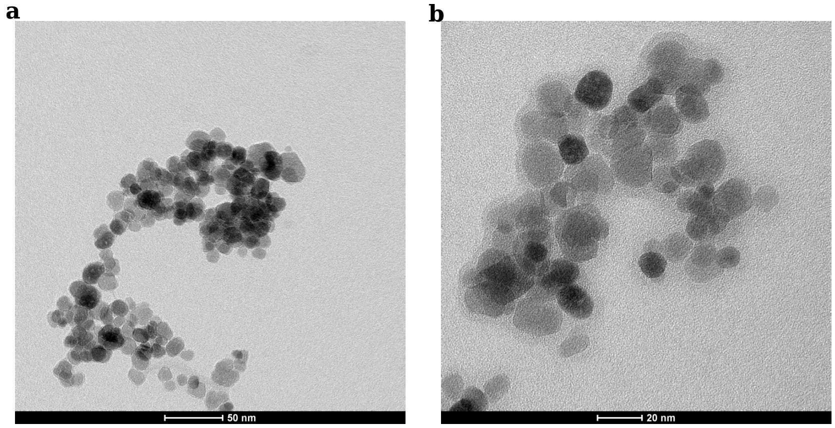

Figure S3 : **Transmission electron microscope (TEM) images of Fe_3O_4 magnetic nanoparticles coated with APTES.**

Complementary to the theoretical model - results for magnetic liquid droplet on stretched LDPE film.

The plots in Figure S4 represent theoretical model results for the number of nanoparticles N per unit segment of the volume "edge" (cf. the Main text of our work) along the ring structure that is left by the drying droplet after the contact line jumps to a new location with a smaller value of radius R. The two cases which have been shown in the figure, when the droplet of magnetic liquid dries without the presence of the external magnetic field and when a magnet (B_r=1T) is 0.2 mm below the droplet. It is directly shown that number of magnetic nanoparticles which are pinned at the contact line is almost twice smaller in the presence of the magnetic field than without the field. The plots also show the effect of different roughness sensed by the outward nanoparticle flow for the flows in different radial directions. This result coincides with the experimental observation in Figure S1 of the smaller number on nanoparticles in the direction perpendicular to the LDPE stretching direction.

Complementary to the theoretical model - contact angle time dependence

Theoretical model results suggest that when the contact line becomes pinned by magnetic nanoparticles the value of the contact line θ decreases and this decrease depends strongly on the value of the external magnetic field B from the magnet below the droplet, above the droplet or if there is no the magnet present.

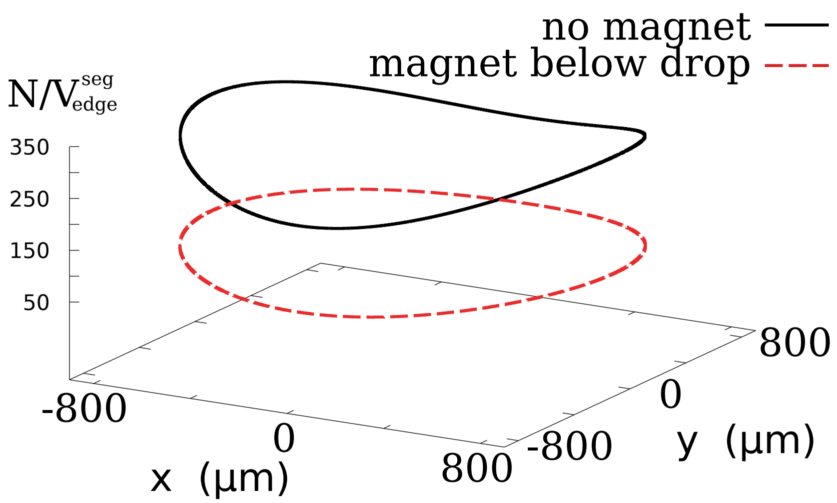

Figure S4 : **Theoretical model results for number of magnetic nanoparticle in a unit segment "edge" in the first nanoparticle ring deposit that has appeared both in the case without and with the magnet.** The x-direction is the LDPE film stretching direction.

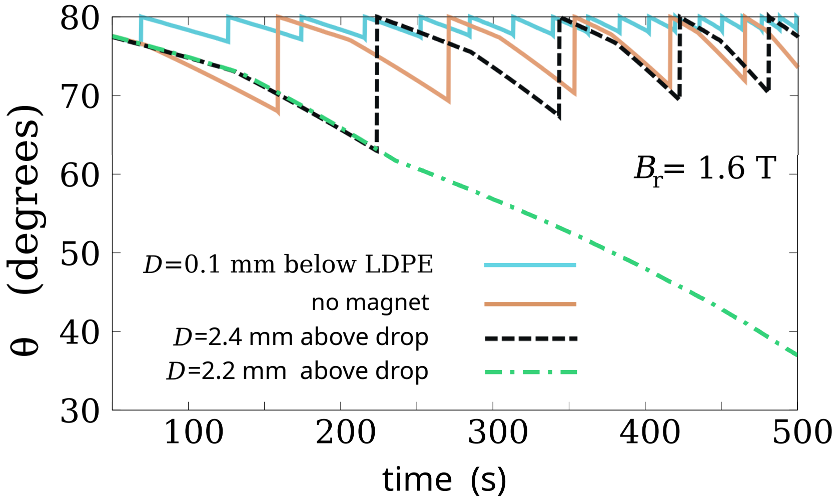

Figure S5 : **Dependence of the contact angle on time for the case shown in Fig.6 in the main text of the work.**
